# The Association of Self-Esteem with the Level of Independent Functioning and the Primary Demographic Factors in Persons over 60 Years of Age

**DOI:** 10.3390/ijerph19041996

**Published:** 2022-02-10

**Authors:** Dorota Ryszewska-Łabędzka, Sławomir Tobis, Sylwia Kropińska, Katarzyna Wieczorowska-Tobis, Dorota Talarska

**Affiliations:** 1 Department of Nursing, Stanislaw Staszic State University of Applied Sciences, 64-920 Pila, Poland; d.labedzka69@gmail.com; 2 Department of Occupational Therapy, Poznan University of Medical Sciences, 60-781 Poznan, Poland; stobis@ump.edu.pl; 3 Geriatric Unit, Department of Palliative Medicine, Poznan University of Medical Sciences, 61-245 Poznan, Poland; skropins@ump.edu.pl; 4 Department of Preventive Medicine, Poznan University of Medical Sciences, 60-781 Poznan, Poland

**Keywords:** self-esteem, the elderly, functional state, care needs

## Abstract

Self-esteem reflects the way we see ourselves. The aim of this study was to determine the relationship among self-esteem, bio-psycho-social functioning, and sociodemographic conditions in the elderly. The study included 300 individuals over 60 years of age living in their home environment. The employed research tools included the Abbreviated Mental Test Score, Rosenberg Self-Esteem Scale (RSES), and EASYCare Standard 2010 questionnaire involving the following scales: independence score, risk of breakdown in care, and risk of falls. Results: The average score achieved by the study group according to the RSES scale was 29.9 ± 5.6 points. In addition, the study group presented a low risk of independence loss (independence score 13.3 ± 18.1), risk of breakdown in care (4.4 ± 2.4), and risk of falls (1.8 ± 1.6). The conducted multivariate analysis demonstrated that a significant (*p* < 0.05) negative predictor of low self-esteem was education below the secondary level, a poor financial condition, and functional limitations in domain I (seeing, hearing, and communicating skills) of the EASYCare Standard 2010 questionnaire. A relationship was found between self-esteem and the level of bio-psycho-social functioning, as well as between education and the financial situation. The results demonstrate that even successfully ageing individuals require a regular assessment of their functional status and individually adapted support in order to maintain independence and to increase their self-esteem.

## 1. Introduction

Self-esteem is defined as the subjective assessment of one’s own worth. It includes the feeling of self-acceptance and a positive attitude towards oneself [1,2,3]. In fact, the general feeling of self-esteem is referred to as global and is contrasted with specific self-esteem, which relates to the assessment of a person’s involvement in various areas of life, such as in social or professional roles. Global self-esteem constitutes a unidimensional concept affecting specific self-esteem [4]. Self-esteem can be analysed both in terms of a trait and a state. As a trait it is characterised by a stable evaluation of oneself, whereas as a state it is based on a specific context, and thus can change due to situational factors [5].

A person develops a sense of self-esteem as a result of positive and negative experiences in life. The greatest increase in self-esteem occurs during childhood and adulthood, and it reaches its peak at approximately 60–70 years of age, and gradually declines with age [1,6]. This may stem from poorer physical and mental functioning, deterioration in social status, financial situation, and the loss of loved ones [7].

In contrast, the elderly tend to accept their functional limitations, which may be reflected in a more positive perception of oneself [6]. A similar effect is frequently observed in individuals who reject stereotypes regarding old age and view themselves more positively. These individuals tend to accept changes in appearance and functional limitations and are willing to undertake new life challenges and participate in social life [8,9]. Is it worth noting that the elderly who are self-confident present higher self-esteem and are more capable of coping with psychosocial issues. On the other hand, individuals who are dissatisfied with their lives or have negative past experiences are less confident, and they view themselves less favourably [10]. Additionally, self-esteem is a significant element in the adaptation processes, and it does not depend on age but on one’s level of social integration and ability to manage challenging life situations [7,11,12].

Positive self-esteem also provides the basis for the development and maintenance of mental health [9,13]. Moreover, it is a vital element in health promotion, as it is conducive to well-being and a sense of happiness [8,10,12]. It also influences better functioning in emotionally difficult situations, such as illness or isolation during a pandemic. A low self-esteem, on the other hand, results in withdrawal from new activities. Additionally, it can be a risk factor for the development of depression, anxiety, and eating disorders as well as for the formation of inappropriate defence mechanisms, such as acts of violence, addiction to alcohol, or psychoactive drugs [12,13,14,15].

Many factors determine the development of self-esteem, and those most commonly identified include a reduced physical capacity and mental ability, increase in symptoms due to comorbidities, gender, ethnicity, socioeconomic status, social relationships, stressful life events, retirement, and the need for gait stabilisation equipment, as well as the loss of a spouse and incidents of previous falls [1,7,10,12,16,17,18,19,20,21]. Other significant elements include respect and attention from loved ones, including family members, and a sense of meaning in life [16,22,23].

The elderly’s perception of themselves and the ageing process affects their assessment of the quality of life [8,9,20,24]. Furthermore, both the assessment of self-esteem and the quality of life depends on the level of the independent fulfilment of needs [8,9,25,26,27]. Another common problem of the elderly is disability. In old age, apart from the changes associated with the aging process itself, the exacerbation of symptoms of chronic diseases is observed. Consequently, the number of persons requiring support increases as well [28,29], although an increasing number of elderly individuals are active and classified as part of the successful ageing group. Therefore, the organisation of care for the elderly that represents a major logistical and financial challenge for both the state and the family. The variety of problems in groups of senior citizens, as well as their varying expectations, indicate the necessity of a multifaceted approach when planning support, i.e., the cooperation of the healthcare system, social care, regional programmes, and cooperation with the elderly and their carers. Hence, since independence is one of the factors promoting high self-esteem, the support provided should promote actions to improve and create environments conducive to maintaining the independence of the elderly. Such measures will contribute to reducing health care costs and to improving the quality of life of the elderly [10,20].

It is vital to bear in mind that functional limitations in the elderly usually develop gradually. Therefore, periodic monitoring of their health status and functional ability allows for the appropriate support, and thus maintains the independence of this group until they reach an older age. One of the tools recommended for monitoring functional ability is the EASYCare Standard 2010 questionnaire [30,31]. It facilitates the assessment of various activities, which definitely constitutes an advantage of this tool, since the level of human functioning is important not only biologically, but also psychosocially. Most studies conducted in the elderly indicate the impact of physical and mental health, social relations, physical activity, or place of residence (community, nursing home) on self-esteem. However, little research has been conducted investigating the relationship between the level of self-satisfaction of biological, psychological, and social needs and self-esteem. Therefore, the aim of our study was to determine the associations among self-esteem, the level of the bio-psycho-social functioning, and the sociodemographic conditions in the elderly.

## 2. Materials and Methods

### 2.1. Organization of the Study

The cross-sectional study was conducted between October and November 2019. The research location was a family doctor’s outpatient clinic (GP), where a family doctor and a community nurse provided free medical care as part of state-funded health insurance. Participation in the study was offered to anyone who received medical advice during the study period and was at least 60 years old. During registration, patients were informed about the conducted study, and they were asked whether they agreed to participate. The questionnaire was filled in by the subjects following an appointment with a GP, in a separate room, in the presence of a nurse. The participants mostly completed the questionnaire on their own. Nurses assisted 10 subjects, who had forgotten their glasses, with reading and completing the questionnaire. The participation in the study was voluntary, and the study initially included 316 participants. Subjects who provided verbal consent were asked to complete the part of the questionnaire containing questions referring to the inclusion and exclusion criteria.

The inclusion criteria were as follows: 1. age ≥ 60 years; 2. residence in a house or a block of flats; and 3. the ability to communicate. The exclusion criteria were as follows: 1. cognitive impairment, on the basis of the AMTS (Abbreviated Mental Test Score) scale. Individuals who scored 7 points or more, which indicated they were cognitively fit, were qualified for further study; and 2. a disease process or disability which prevents independent functioning. The study group scored 9.9 ± 0.5 points (median: 10; range 7–10 points) on the AMTS scale.

Finally, 300 individuals were included in the study and completed research questionnaires labelled with an identification number. Fourteen persons were excluded from the study, as they did not meet the inclusion criteria, and 2 persons opted out on their own in the course of completing the questionnaires.

Prior to the distribution of the questionnaires, the nurses reminded the subjects once again of the voluntary character of their participation in the study. Additionally, a declaration was provided on the first page of the questionnaires, in which the patient stated that participation in the study was voluntary and that they had read the questionnaires.

### 2.2. Research Tools

1. AMTS scale (Abbreviated Mental Test Score, Hodkinson, 1972) [32]—used for the screening assessment of cognitive function. The scale ranges from 0 to 10 points. The score indicating normal mental performance is between 7 and 10 points. This tool was employed only when verifying the group, in order to evaluate the inclusion criterion.
2. Rosenberg Self-Esteem Scale (RSES).

The RSES scale according to the Polish edition by Łaguna et al. was applied [18]. The validated questionnaire for the Polish population demonstrated good psychometric properties (reliability and validity). It consists of 10 statements to which the participant responds by choosing 1 of 4 possible statements, according to the Likert scale ranging from: 1—strongly agree to 4—strongly disagree. Thus, the range of scores for the entire scale is from 10 to 40 points. In the Polish version of the score, the scores for items 1, 2, 4, 6, and 7 should be reversed. The general assessment principle assumes that the higher the final score, the higher the feeling of self-esteem.

Authors of the previous papers interpreted the results of the RSES scale differently [2,3,10,12,14,24]. In our study, we used the following scoring: low self-esteem was indicated by 10–27 points and high self-esteem by 28–40 points, taking into account the median (28 points) and 60% of the obtained score on the axis of 10–40 points.

3. EASYCare Standard questionnaire 2010.

The EASYCare Standard questionnaire 2010 was implemented to assess independent functioning. The tool is recommended for the elderly when assessing care needs (need for support) [30,31]. The Polish version of the tool was adapted by Bień et al. [33]. The questionnaire consists of two parts. Firstly, information regarding the patient and his family situation is collected, and secondly, the level of independent functioning is assessed in 7 domains: I: seeing, hearing and communicating (4 items); II: looking after yourself (13 items); III: getting around (8 items); IV: your safety (5 items); V: your accommodation and finance (3 items); VI: staying healthy (7 items); and VII: your mental health and well-being (9 items).

On the basis of the collected information, a list of the identified caring needs is established, reflecting the activities in which a person is not independent. In these domains the elderly require support, and hence they become priorities when organizing care.

The last summarizing element are the following three scales providing information from the previously analysed domains:

- Independence score—indicates the independence of a person in terms of dressing, caring for appearance, bathing, preparing meals, moving around independently, and managing finances. The number of points that can be received is 0–100 points. The higher the number of points received, the greater the risk of dependence.
- Risk of breakdown in care—reflects the risk of hospitalization on the basis of the obtained answers concerning such activities as dressing, bathing, using the toilet, and assessing health and well-being, as well as memory loss, depression and/or pain. Scoring range is 0–12 points. With an increase in the number of points, the risk of 24 h care increases.
- Risk of falls—is evaluated on the basis of the analysis of the difficulties in movement, foot problems, and feeling of safety at home and outside, as well as the number of falls in the last 12 months. Scoring range is 0–8 points. Scoring 3 or more points indicates a high risk of falls.

### 2.3. Statistical Analysis

The comparison of independence score, risk of brake down, and risk of falls between two groups of patients (60–69 years vs. 70 and above) was performed by the Mann–Whitney test since data did not follow the normal distribution (Shapiro–Wilk test). The categorical data, including age, marital status, place of residence, education, form of residence, requiring a carer, and low or high self-esteem, were compared using the chi^2^ test for independence. Spearman’s rank correlation coefficient was used to analyse the relationship between the results of the EASYCare questionnaire and the RSES scale.

In addition, a univariate and multivariate logistic regression analysis was conducted to determine the effects of demographic and social variables on the functioning of the elderly in each of the EASYCare Standard 2010 domains and the risk scales, as well as on the level of self-esteem (RSES). For the multivariable logistic regression, a stepwise backward selection procedure was used. All the tests were considered significant at *p* < 0.05.

Statistical calculations were performed using TIBCO Software Inc. (2017; Palo Alto, CA, USA), Statistica version 13.

## 3. Results

### 3.1. Characteristics of the Studied Group

The study involved a group of 300 individuals (see Table 1), where almost 2/3 were women (64.7%). The average age was 70.5 ± 7.5. The majority (54.7%) of the subjects in the group were aged 60–69 years. Additionally, the majority (80.0%) lived in the city and had completed secondary education (40.7%). The married couples accounted for 59.3% of the group. A total of 52.6% of the women and only 18.9% of the men had no life partner. A total of 26.7% of the subjects lived alone. A total of 15.7% of participants provided care for another person, which concerned women more often. Only about ¼ (24.0%) of the group benefited from the care of other persons. The majority (61.7%) reported they were in good financial condition.

### 3.2. Self-Esteem in the Studied Group

On the Rosenberg Self-Esteem Scale (RSES), the study group scored 29.9 ± 5.6 points, which indicates an average self-esteem according to the adopted distribution. A low self-esteem was found in 23.3% of the group.

Following the analysis of the demographic and social variables, significant differences in low self-esteem were demonstrated only with such predictors as education with 6.3% having a secondary and higher education level vs. 17.0% having an education below the secondary level (*p* < 0.001), place of residence with 6.0% living in urban areas vs. 17.3% living in rural areas (*p* < 0.025), financial condition with 15.7% having a good financial condition vs. 35.7% with a poor financial condition (*p* < 0.007), and requiring a carer with 43.0% requiring a carer vs. 17.1% not requiring a carer (*p* < 0.001). Individuals whose education was below the secondary level, living in the rural areas, having a poor financial condition, and requiring the support of a carer presented lower self-esteem. The analysis of individual questions from the RSES scale with demographic and social variables revealed a difference in self-esteem assessment in all questions regarding education and requiring a carer.

### 3.3. Functional Level of the Studied Group—EASYCare Standard 2010 Questionnaire

**Figure 1:**
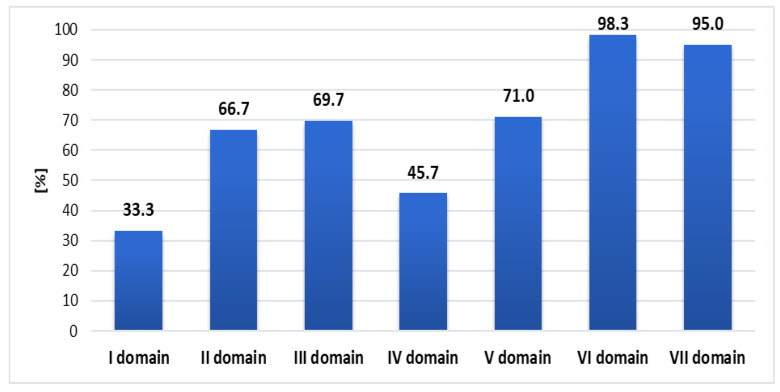
The percentage of needs (lack of independence) in particular domains. I: seeing, hearing, and communicating (4 items); II: looking after yourself (13 items); III: getting around (8 items); IV: your safety (5 items); V: your accommodation and finance (3 items); VI: staying healthy (7 items); and VII: your mental health and well-being (9 items).

Out of the seven domains of the EASYCare Standard 2010 questionnaire, the majority of subjects reported limitations in independent functioning in domain VI—staying healthy and VII—your mental health and well-being, whereas the least number of subjects reported limitations in domain I—seeing, hearing, and communicating (see Figure 1).

In domain VI (see Table 2), the limitations resulted mainly from the following factors: 60% (*n* = 180) complained of rapid fatigue during daily work, 44.7% (*n* = 134) were affected by overweight, 42.0% (*n* = 126) of subjects reported no regular physical activity, and 86.0% (*n* = 258) of subjects were not aware of the currently required vaccinations. This factor was the main contributor to the unmet needs in domain VI and, consequently, this area was found to require the most support.

In the last domain, VII, 66.3% (*n* = 199) of the studied group reported memory issues (forgetting), 63.0% (*n* = 189) experienced loneliness, 65.3% (*n* = 196) presented problems sleeping, and 75% (*n* = 225) were in pain. Additionally, 1/3 of the group experienced depression and a lack of interest. In the remaining domains the problems were caused by such elements as in domain I; most difficulties resulted from hearing impairment and the inability to use the phone (14.3%); in domain II, 27.3% (*n* = 82) of participants complained of difficulties with performing housework, whereas 53.7% (*n* = 161) reported problems with urine incontinence and 43.7% (*n* = 131) with bowel movements.

In domain III half (50.0%, *n* = 150) of the subjects complained of problems with feet, whereas 43.7% (*n* = 131) experienced falls in the last 12 months, and 28.7% (*n* = 86) of participants were unable to go shopping by themselves.

In terms of personal safety (domain IV), 37% (*n* = 111) of subjects indicated that they did not feel safe outside their homes. In domain V, your accommodation and finance, 67.7% (*n* = 203) of participants wished to obtain information regarding financial assistance.

The resulting limitations in individual domains have been reflected in the three scales summarizing the EASYCare Standard 2010 questionnaire. The average scores in the three summary scales were: independence score—13.3 ± 18.1, risk of breakdown in care—4.4 ± 2.4, risk of falls—1.8 ± 1.6. The results confirm the high functionality of the group.

Taking into consideration the demographic variables, significant differences (*p* < 0.05) were found between independence in the three summary scales and age, marital status, education, and requiring a carer. No difference (*p* > 0.05) was observed with regard to place and form of residence. The analysis of age according to the groups demonstrated that with age, the efficiency of the elderly in performing independent care activities decreases, which was reflected in the results of the three scales summarizing the questionnaire EASYCare Standard 2010.

### 3.4. Rosenberg Self-Esteem Scale vs. Questionnaire EASYCare Standard 2010

The Spearman rank correlation coefficient R_s_ demonstrated a correlation between self-esteem (RSES) and the functional level of the elderly in all domains and three EASYCare Standard 2010 summary scales. The following results were obtained: with an increased risk of dependence (independence score rs = −0.295, *p* < 0.001), increased risk of breakdown in care rs = −0.453, *p* < 0.001), increased risk of falls (risk of falls rs = −0.301, *p* < 0.001), and lower self-assessment scores were observed.

### 3.5. Rosenberg Self-Esteem Scale vs. Questionnaire EASYCare Standard 2010 and Demographic and Social Variables

Demographic factors were selected for the logistic regression univariate model (see Table 3), as well as the domains and summary scales of the EASYCare Standard 2010 questionnaire, which scored *p* < 0.05 in the earlier analysis using RSES. All factors were found to be significant, except for the place of residence. It should be emphasized that in domains VI and VII the majority of subjects reported unmet needs, which resulted in statistically insignificant results in the analysis (*p* > 0.05).

The conducted multivariate analysis (see Table 3) demonstrated that a low self-esteem was at least three times more common among individuals with an education below the secondary level and more than twice as often in subjects with a poor financial condition. Furthermore, participants who reported no needs in domain I (seeing, hearing, and communicating skills) were three times less likely to experience low self-esteem.

## 4. Discussion

The majority of study participants were aged 60–69 years. Most of them lived with their spouse or children in urban areas, their financial condition was good, and they had at least been achieved secondary education. Good cognitive functioning was confirmed with the AMTS scale in all subjects. Additionally, most of the participants presented high self-esteem (29.9 points). However, the RSES score only slightly exceeded the threshold we adopted. A high self-esteem in the elderly has also been reported by other authors, although they simultaneously emphasise that it decreases with age [1,17,24]. The changing roles may account for this phenomenon, particularly bearing in mind elements such as children moving out, retirement, poorer financial situation, and deteriorating health and also a reduced participation in family life, or insufficient social support [3,6,34].

Although the participants demonstrated a high level of independence in the EASYCare Standard questionnaire 2010 and, therefore, a low risk of disability (independence score), institutional care (risk of breakdown in care scale) or falls (risk of falls scale), they also reported a relatively high number of needs requiring support. Moreover, the elderly expected most assistance in area VI (staying healthy) and VII (your mental health and well-being) included in the EASYCare Standards 2010 questionnaire. In terms of the needs, the elderly most frequently indicated the need for pain relief (2/3 of the group), improving their knowledge about current vaccinations, and the possibilities of financial support. The importance of vaccinations in the elderly was already emphasised in 2010 by the experts of the European Union Geriatric Medicine Society and the International Association of Gerontology and Geriatrics European Region. The most commonly recommended vaccinations include: influenza, tetanus, diphtheria, pertussis, pneumococcus, and shingles [35]. Furthermore, the recently developing pandemic has prompted an additional recommendation for vaccination against COVID-19 (coronavirus disease 2019), particularly in the elderly.

Other issues reported by more than 50% of the group were: experiencing shortness of breath while doing housework, sleep disorders, incontinence, foot problems, and overweight. All of the aforementioned disorders are commonly identified as factors limiting the independence of the elderly and deteriorating their self-esteem [26,27]. In area VII, the subjects particularly highlighted forgetfulness, as well as feelings of despondency and loneliness. In fact, the feeling of loneliness and isolation frequently accompanies the elderly and adversely affects their self-esteem, reducing their quality of life [12,24,36]. In addition, loneliness can also lead to serious health issues [18]; therefore, it is crucial for the elderly to maintain interpersonal relationships, particularly with family [3,12,15,16,23]. The level of independent functioning largely depends on mood. In the study group, lowered mood was present in 1/3 of the group and was accompanied by problems, such as forgetfulness, feelings of loneliness, sleep disorders, and chronic pain. It is vital to bear in mind that lowered mood, particularly depression, is a factor significantly associated with low self-esteem [12,13,15,16].

In the conducted study, the independent correlates of low self-esteem were education below the secondary level, having a carer, a poorer financial condition, and unmet needs in Area I: seeing, hearing, and communicating and IV: your safety. According to Tavares et al. [24], sensory functions affect the quality of social interactions. They allow for interaction with their environment and also influence a person’s independence. Therefore, a deterioration of vision, hearing, or speech fluency disorders all promote low self-esteem.

Rosenberg and Pearlin remarked as early as 1978 that poorer education is associated with poorer self-esteem [10]. It is believed that higher education is one of the factors which allow us to manage the challenges posed by the aging process more effectively [12]. Additionally, it also promotes a better financial condition, which plays a significant role in self-perception [3,8,20].

The logistic regression—univariate model additionally revealed a significant association between low self-esteem and the need for a carer. A total of 24% of the study participants received care from others. This group included individuals who were less functional and required support in daily activities. According to a study conducted in the UK, approximately 20% of men and 30% of women over 65 years of age require assistance with at least one activity of daily living (ADL) [29]. The most common sources of disability include ailments resulting from chronic diseases and comorbidities. In turn, functional limitations and the experienced ailments negatively affect mood and self-esteem [1,19]. In contrast, coping with the disease is also influenced by self-compassion, which is a feeling facilitating the acceptance of pain and other disorders or imperfections allowing one to take care of oneself. It is particularly important in the elderly, as disorders increase and the functional capacity decreases with age. High self-esteem positively affects self-compassion [13]. The ability to function independently is frequently indicated as a major determinant of a high quality of life and self-esteem [14,25,26,27]. In contrast, poor independence decreases self-esteem [2,20,21].

The results obtained in the conducted study demonstrate that age represents an important determinant of caregiving needs, and that despite good functional ability, the elderly require support on the part of others. Therefore, when planning care for the elderly, both nationally and individually, particular attention should be paid to the appropriate choice of support to extend independent functioning [37]. It is also essential to increase the activity of the elderly in all areas of life [12,18], as well as to take into account the individual differences in the functioning of elderly persons, and not to focus only on the disabled. The abovementioned measures will contribute not only to increasing the independence of the elderly, but also to their increased self-esteem and a better quality of life [8,10].

Measures aimed at increasing self-esteem in the elderly include:

1. Developing social connections. The elderly surrounded by family and friends present higher self-esteem. 2. Eliminating negative stereotypes. Positive attitudes towards the elderly motivate them to take on new challenges in life. 3. Exploring the views and opinions of the elderly. This offers insight into their perception of current events and provides information regarding changes in their environment. 4. Using the experience and knowledge of the elderly. 5. Adapting the accommodation to the degree of disability and ensuring safe mobility. Physical activity helps prevent falls and maintains independent functioning.

Good self-esteem facilitates understanding and acceptance of the ageing process and thus should be taken into account when organising care for the elderly [3]. Moreover, providing appropriate support is possible when monitoring the elderly’s functional status and self-esteem. However, in order to do so, appropriate programmes and legislation need to be implemented.

### Study Limitations

The study included fit individuals over 60 years of age. In the future, the study group should be extended to include subjects at a significantly lower functional level and in greater need of support. This would possibly demonstrate that some care needs increase with age, whereas other needs increase with disability. First of all, increasing the group size would allow for the identification of subgroups and a more accurate analysis of changes in self-esteem, e.g., separately for younger and older men, or women, as well as for individuals with a higher or lower functionality level. Secondly, the study involved a subjective assessment. Although we used the reliable RSES and EASYCare Standard 2010 tools, a verification of the actual functioning at home would certainly have produced more objective results. Finally, in the statistical analysis, in terms of the predictors affecting self-esteem, we did not include the number of diseases or persistent complaints.

## 5. Conclusions

The results confirm that even successfully ageing individuals require regular assessment of their functional status and individually adapted support in order to maintain independence. Therefore, scales to determine the need for support, e.g., the EASYCare Standard 2010, should be introduced and obligatorily used by community nurses and social services.

Multivariate logistic regression found a relationship between low self-esteem and a poor financial condition, poor education, and sight, hearing, and communication problems. Furthermore, the predictors of low self-esteem revealed the specificity of activities to be included in programmes aimed at the elderly: 1. regular check-ups of sight and hearing and fitting of suitable prostheses; 2. preparing and providing the elderly with information on the possibilities of financial and material support; and 3. providing access to and encouraging participation in various forms of lifelong learning.

Nevertheless, in order to achieve the objectives of such a programme, it is essential to develop and implement a comprehensive strategy aiming to improve the quality of life of the elderly and to increase their self-esteem.

## Figures and Tables

**Table 1:** Characteristics of the studied group—demographic variables.

Variable	Classification	Total N (%)
Gender	Entire group	300 (100.0)
	Women	194 (64.7)
	Men	106 (35.3)
Age	Entire group	70.5 ± 7.5
	60–69 years of age	164 (54.7)
	Over 70 years of age	136 (45.3)
Place of residence	Rural areas	60 (20.0)
	City	240 (80.0)
Marital status	Single	21 (7.0)
	Married	178 (59.3)
	Divorced	9 (3.0)
	Widow/Widower	92 (30.7)
Education	Elementary	54 (18.0)
	Vocational	86 (28.7)
	Secondary	122 (40.7)
	Higher	38 (12.7)
Form of residence	Single	80 (26.7)
	As a couple	115 (38.3)
	In a family	105 (35.0)

**Table 2:** The most frequent limitations in EASYCare Standard 2010 questionnaire.

EASYCare Domain	Study Group N (%)	Number of Subjects Reporting Needs in the Domain		*p* Value
		60–69 Years of Age N (%)	Over 70 Years of Age N (%)	
I: Seeing, hearing, and communicating (4 items)	100 (33.3%)	38 (23.2%)	62 (45.6%)	<0.001 *
II: Looking after yourself (13 items)	200 (66.7%)	91 (55.5%)	109 (80.1%)	<0.001 *
III: Getting around (8 items)	209 (69.7%)	98 (59.8%)	111 (81.6%)	<0.001 *
IV: Your safety (5 items)	137 (45.7%)	65 (39.6%)	72 (52.9%)	0.027 *
V: Your accommodation and finance (3 items)	213 (71.0%)	116 (70.7%)	97 (71.3%)	1.000 *
VI: Staying healthy (7 items)	295 (98.3%)	160 (97.6%)	135 (99.3%)	0.049 *
VII: Your mental health and well-being (9 items)	285 (95.0%)	157 (95.7%)	128 (94.1%)	0.600 *
Summary scales				
Independence score	13.3 ± 18.1	8.8 ± 14.5	18.6 ± 20.6	0.000 **
Risk of breakdown in care	4.4 ± 2.4	4.1 ± 2.4	4.9 ± 2.4	<0.001 **
Risk of falls	1.8 ± 1.6	1.4 ± 1.5	2.2 ± 1.7	<0.001 **

* chi^2^ test; ** Mann–Whitney test.

**Table 3:** Logistic regression—univariate model and multivariate model for self-esteem (Y = 1—low; Y = 0—high self-esteem).

Logistic Regression	Univariate Model				Multivariate Model			
Analysed Domain (Reference Level)	OR	95% CI		*p*-Value	OR	95% CI		*p*-Value
Age (60–69 years of age)	0.73	0.43	1.24	0.242				
Gender (women)	1.21	0.70	2.09	0.481				
Place of residence (city)	0.65	0.34	1.21	0.172				
Marital status (single)	1.21	0.70	2.09	0.481				
Form of residence (alone)	0.53	0.23	1.24	0.136				
Education (below the secondary level)	4.25	2.36	7.67	<0.001	3.54	1.91	6.56	<0.001
Financial condition (poor financial condition)	2.98	1.72	5.17	<0.001	2.49	1.28	4.31	<0.010
Requiring a carer (yes)	3.66	2.05	6.55	<0.001				
Place of residence (living in the city)	0.65	0.34	1.21	0.172				
I (Lack of need)	0.27	0.15	0.46	<0.001	0.29	0.17	0.54	<0.001
II (Lack of need)	0.52	0.28	0.96	0.034				
III (Lack of need)	0.50	0.26	0.95	0.032				
IV (Lack of need)	0.24	0.17	0.44	<0.001				
V (Lack of need)	0.25	0.11	0.54	<0.001				
VI (Lack of need)								
VII (Lack of need)	0.22	0.29	1.73	0.077				
Independence score (Independence)	0.31	0.18	0.54	<0.001				
Risk of breakdown in care (Risk)	3.85	2.17	7.14	<0.001				
Risk of falls (point 1–2)	0.20	0.12	0.36	<0.001				

OR—odds ratio, 95% CI—confidence interval.

## Data Availability

The datasets used during the current study are available from the corresponding author on reasonable request.
